# Implications of lysyl oxidase-like protein 3 expression in the periodontium of diabetic rats

**DOI:** 10.1590/1678-7757-2022-0176

**Published:** 2022-09-19

**Authors:** Li HUANG, Jun CHEN, ZUO Yuling, LI Jinle, YUE Yuan, Min WANG, HAO Liang

**Affiliations:** 1 West China Hospital of Stomatology National Clinical Research Center for Oral Diseases Sichuan University Chengdu China West China Hospital of Stomatology, National Clinical Research Center for Oral Diseases, Sichuan University, State Key Laboratory of Oral Diseases, Chengdu, China.; 2 Chengdu University Chengdu China Hospital of Chengdu University of Traditional Chinese Medicine, Chengdu, China.; 3 Northwest Minzu University Key Laboratory of Oral Diseases of Gansu Province Key Laboratory of Stomatology of State Ethnic Affairs Commission Lanzhou China Northwest Minzu University, Key Laboratory of Oral Diseases of Gansu Province/Key Laboratory of Stomatology of State Ethnic Affairs Commission, Lanzhou, Gansu, China.

**Keywords:** Lysyl oxidases, Periodontal ligament, Diabetes, Periodontal diseases, LOXL3

## Abstract

**Objectives:**

Diabetes has been strongly associated with periodontal diseases. The periodontal ligament (PDL) has an abundant extracellular matrix (ECM). Lysyl oxidases (LOXs) are closely associated with various diseases caused by abnormal ECM functions, however, the role of LOXs in periodontal diseases induced by diabetes remains unclear.

**Methodology:**

In this study, 8-week-old Zucker diabetic fatty rats were used to establish a type 2 diabetes mellitus (T2DM) model. After 9 and 16 weeks, hematoxylin and eosin (H&E), Masson’s trichrome, and immunohistochemical staining were performed.

**Results:**

After 9 weeks, loose collagen fibers were found in the interradicular area of the diabetic group, in opposition to the control group. There were no significant differences in LOX expression between the diabetic and control groups (p>0.05). However, after 16 weeks, the diabetic group presented a disordered arrangement of the PDL, showing decreased collagen content and significantly increased lysyl oxidase-like protein 3 (LOXL3) expression when compared with the control group (p<0.05). This suggests that LOXL3 plays a significant role in periodontal histopathological changes in diabetic rats.

**Conclusion:**

Our study showed elevated LOXL3 expression in the PDL of diabetic rats after 16 weeks, suggesting that LOXL3 may be involved in the occurrence and development of periodontal histopathological changes in diabetic rats. LOXL3 could be further used as an indicator for the early diagnosis of diabetic periodontitis in T2DM patients in clinical settings.

## Introduction

Diabetes mellitus (DM) is a global disease that causes severe morbidity and mortality in humans. A large proportion of DM cases is characterized as type 2 DM (T2DM), which is attributed to unhealthy eating habits, obesity, and physical inactivity.^1^ T2DM can lead to complications in various organs, such as the heart,^2^ eyes,^3^ kidneys,^4^ and nervous system,^5^ resulting in high financial and health burden on patients.^6^ T2DM complications are difficult to diagnose at the early stages of the disease because there is a gradual increase in blood glucose levels without obvious and typical clinical symptoms. However, during this early stage of the disease, there is considerable damage to related tissues, which could be detected by histopathological examination.^7^ In clinical settings, T2DM and periodontal diseases have a positive correlation.^8,9^ Patients with more severe diabetes tend to have more serious periodontal diseases and vice versa. Therefore, the histopathological examination of periodontal tissue changes is very significant in the early diagnosis of periodontal diseases induced by diabetes.

Lysyl oxidases (LOXs) are a series of enzymes characterized as copper-dependent enzymes, which can stabilize matrix components. LOXs exist in humans and animals in five different forms: lysyl oxidase (LOX) and lysyl oxidase-like 1–4 (LOXL1–4).^10^ LOXs are potential biomarkers for diseases such as cardiovascular diseases, neurodegeneration, and cancer metastasis.^10^ In our previous studies, abnormal LOX expression was also observed in the kidney tissue and bone matrix of Zucker diabetic fatty (ZDF) rats, suggesting that LOXs play a part in diabetic nephropathy and bone fragility.^7,11^ Moreover, LOXs play an important role in remodeling the extracellular matrix (ECM) by cross-linking collagen and elastin.^12,13^ Vallet and Ricard-Blum^14^ (2019) showed that LOXs are crucial for fiber aggregation and stability, which have been associated with a variety of diseases with abnormal ECM synthesis or degradation, including myocardial fibrosis, atherosclerosis, pulmonary fibrosis, hypertrophic scar formation, and liver hepatic fibrosis.^15^ The periodontal ligament (PDL) has abundant ECM and the metabolism of periodontal tissue depends on the dynamic interaction between PDL cells and the microenvironment provided by ECM.^16,17^ The cross-linking of the ECM is the first step to ensure the maturity and stability of the matrix and this is mediated by extracellular enzymes, including LOXs and transglutaminase.^15^ It can be inferred that LOX-related collagen cross-linking may play an important role in periodontal diseases. Histopathological examination of LOX expressions may be a way to identify periodontal diseases induced by diabetes. However, no related studies have yet been conducted.

In this study, a diabetic ZDF rat model^7^ was established to observe changes in histopathology and LOX expression in the periodontium, and to provide a theoretical basis for early diagnosis of periodontal diseases in T2DM patients in dental clinics.

## Methodology

### Experimental animals

This study was performed in accordance with the guidelines of the Research Ethics Committee of the West China College of Stomatology of Sichuan University (WCCSIRB-D-2015-135). All experiments were performed in the State Key Laboratory of Oral Diseases of Sichuan University, under the established institutional guidelines for the use of experimental animals. The experiments were repeated three times on three different occasions.

### Reagents and instruments

The following reagents and instruments were used: hematoxylin and eosin (H&E) and Masson’s trichrome staining kit (Rongbai Biological Technology Co., Ltd., Shanghai, China); primary antibodies against LOX, LOXL1, LOXL2, and LOXL3 (Abcam, Cambridge, UK); secondary antibody (ABC Kit, Vector Laboratories, California, USA); DAB Kit (Beijing Zhonshan Golden Bridge Biotechnology Co., Ltd., Beijing, China); automatic dehydrator (Leica Co., Germany); paraffin embedding machine (Leica Co., Germany); paraffin slicer (Leica Co., Germany); sliding microtome (Leica Co., Germany); optical microscope and mapping system (Nikon Co., Japan).

### Experimental methods

#### Group and processing

In this study, to establish a T2DM model, 8-week-old obese male ZDF rats (ZDF-Leprfa/Crl, fa/fa) (diabetic group) and lean male ZDF rats (fa/+) (control group) were purchased from Beijing Vital River Laboratory Animal Technology Co., Ltd. (Beijing, China) and fed with a high-fat diet (Purina 5008, Harlan Teklad, Indianapolis, IN, USA). The total number of rats was 60 and rats were randomly divided into two groups, according to the time of sample collection: 9 weeks and 16 weeks (n=10 in each group). All rats were housed at a temperature of 20–25°C and 65–69% humidity under a 12-hour light/dark cycle with free access to food and water. During the whole experiment, the position of the cages was not changed and the physical conditions of rats were observed every other day. Any abnormality found was treated in time.

#### Tissue preparation

At 9 and 16 weeks after induction of diabetes, rats were euthanized and then maxillary samples were extracted. After removing the soft tissue, the maxillary samples were fixed in 4% paraformaldehyde for 48 hours and decalcified for five weeks.

#### Histopathological examination

**Figure 1:**
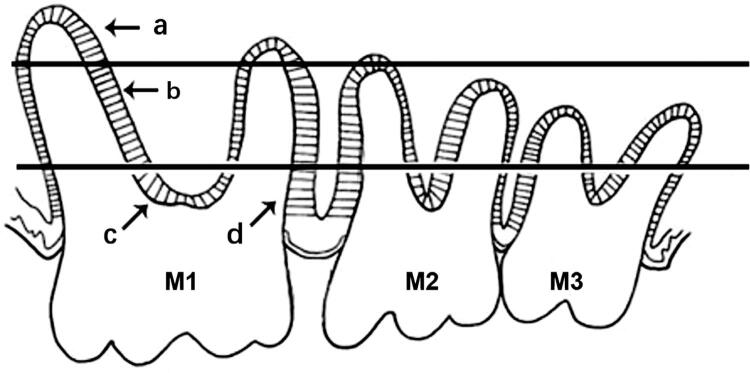
Division of different areas of the periodontal ligament. (M1: first molar; M2: second molar; M3: third molar; a: apical area; b: oblique area; c: interradicular area; d: alveolar crest area)

In this study, the periodontal ligament of the first molar (M1) was divided into four parts. The upper part is the apical area, the middle part is the oblique area, and the lower part is the interradicular and alveolar crest areas (Figure 1). After 9 and 16 weeks, H&E and Masson’s trichrome staining were performed to evaluate periodontal histopathological changes.

#### Immunohistochemical staining

All samples were processed as described in our previous study.^7^ The dilution ratios of the primary antibodies were: anti-LOX (1:400), anti-LOXL1 (1:400), anti-LOXL2 (1:400), and anti-LOXL3 (1:400). The mean integrated optical density (OD) was analyzed using the ImageJ software (National Institutes of Health, Bethesda, MD, USA).

## Statistical analysis

The mean optical density (OD) was considered the analysis index and the OD of LOX, LOXL1, LOXL2, and LOXL3 is presented as mean±standard deviation. The SPSS software (SPSS Inc., USA) was used to compare data between different groups. The Mann–Whitney U test was used for nonparametric data and U>1.96 was considered statistically significant.

## Results

### Histopathological changes in the periodontal tissue

**Figure 2:**
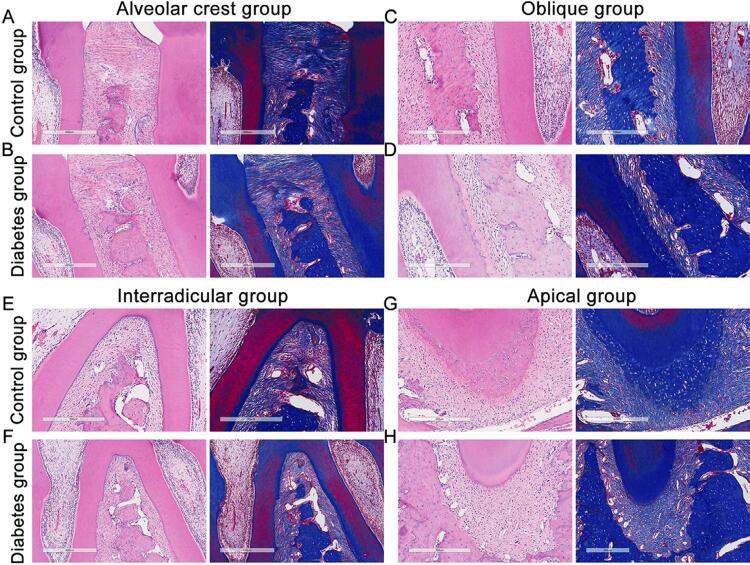
H&E and Masson’s trichrome staining at different parts of the periodontal ligament in ZDF rats after 9 weeks. Collagen fibers are stained blue in the Masson’s trichrome staining. n=5, repeated three times. Scale bar: 300 μm

**Figure 3:**
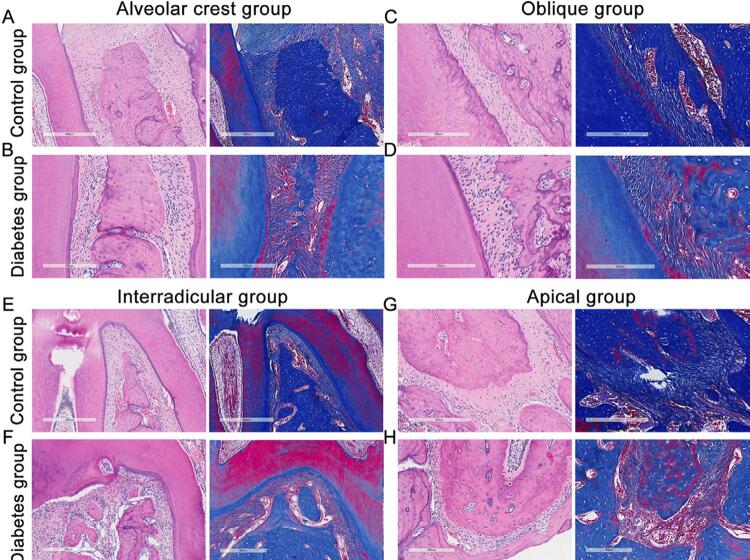
H&E and Masson’s trichrome staining at different parts of the periodontal ligament in ZDF rats after 16 weeks. Collagen fibers are stained blue in the Masson’s trichrome staining. n=5, repeated three times. Scale bar: 300 μm

In this study, histopathological changes were observed by H&E and Masson’s trichrome staining. After 9 weeks, no obvious changes were observed in the control group, however, in the diabetic group, alveolar bone discontinuity and reduction in collagen fiber content (colored in blue) were observed (Figure 2A–H). After 16 weeks, in diabetic rats, except for the visibly decreased content of collagen fibers, the arrangement of periodontal fibroblasts was disordered and the cytoplasm was spindle-shaped with long oval or flat nuclei. Different from the control group, there were several degenerative changes in cells and individual cells were barely recognizable. Moreover, the periodontal ligament lost continuity and showed a disordered arrangement, and alveolar bone resorption was observed (Figure 3A–H).

### LOX expression in the PDL

**Figure 4:**
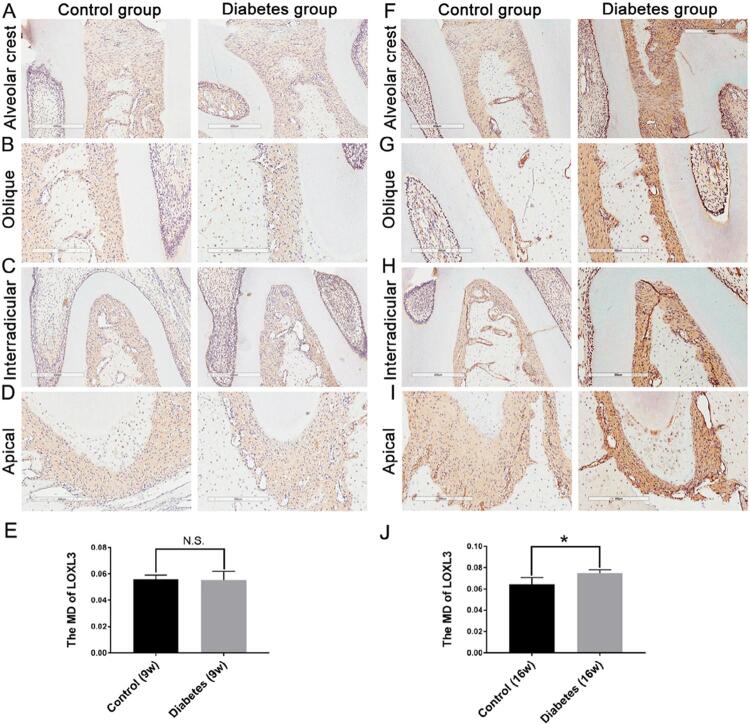
Immunohistochemical observations of LOXL3 in the periodontal ligament of ZDF rats after both 9 and 16 weeks. *p<0.05, in comparison with the control group. n=5, repeated three times. Scale bar: 300 μm

**Supplementary Figure 1:**
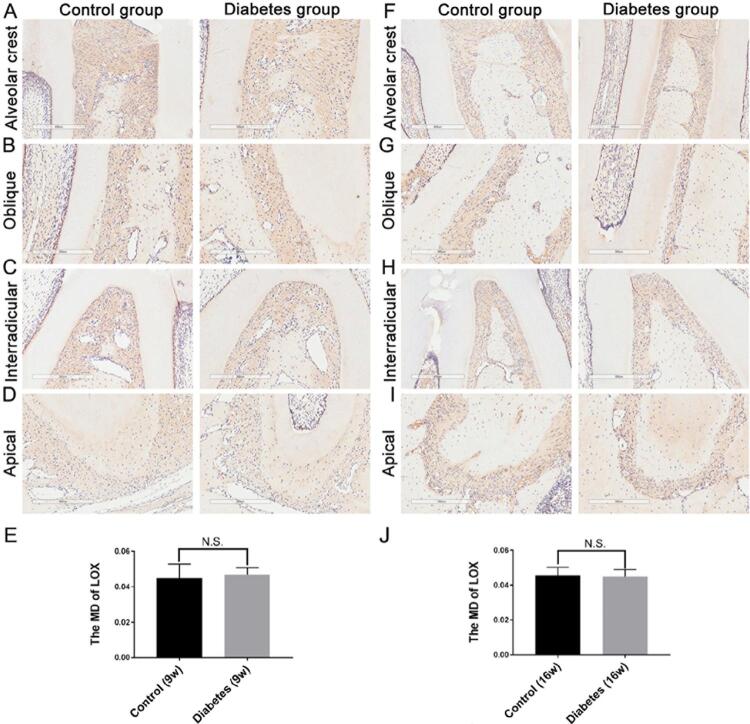
LOX expression in the periodontal ligament

**Supplementary Figure 2 f06:**
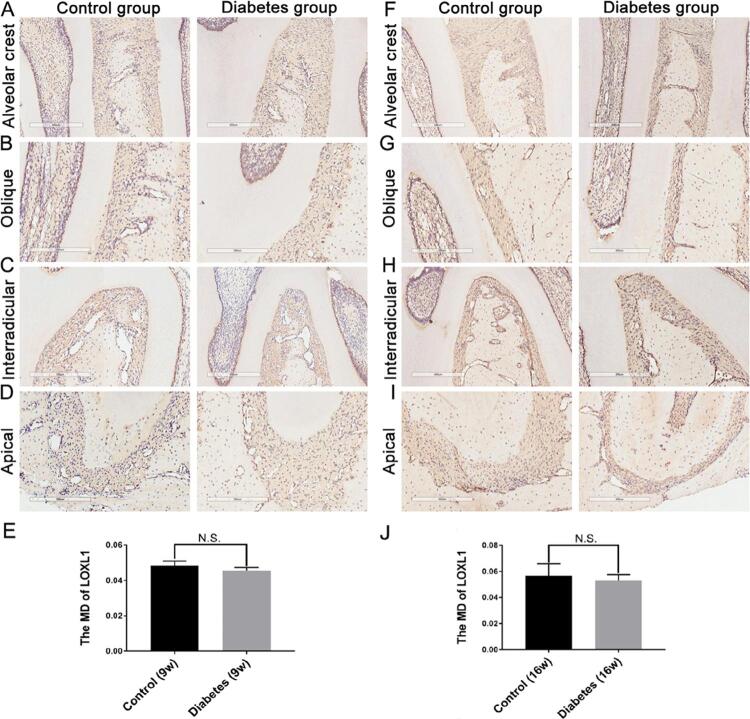
LOXL1 expression in the periodontal ligament

**Supplementary Figure 3 f07:**
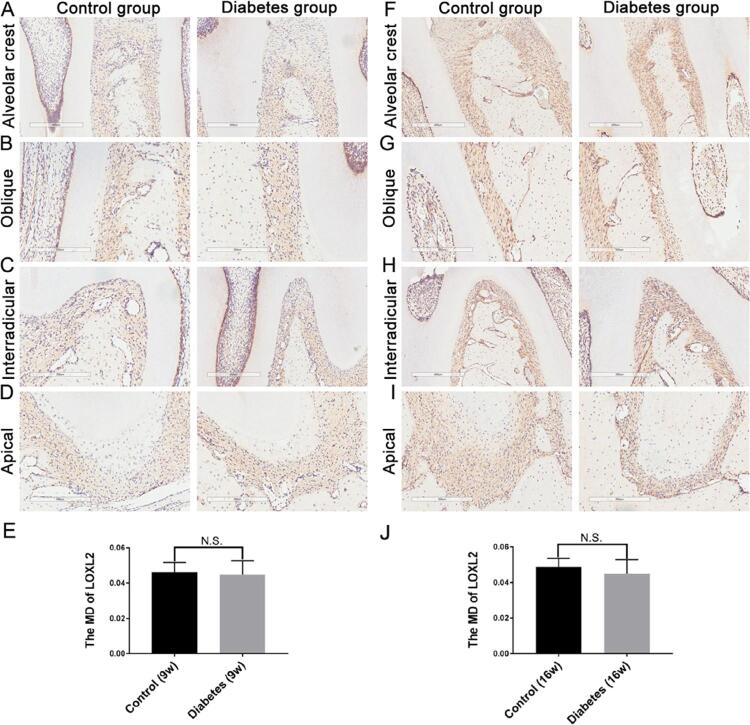
LOXL2 expression in the periodontal ligament

After both 9 and 16 weeks, there were no significant differences in LOX, LOXL1, and LOXL2 expressions between the diabetic and control groups Supplementary Figure 1–3 [Fig f06] [Fig f07]). After 9 weeks, LOXL3 expression in diabetic rats showed a slight increase throughout the PDL (Figure 4A–D), but the difference was not statistically significant (Figure 4E; control group: MD=0.0556+0.0035; diabetes group: MD=0.0551±0.0067; p>0.05). After 16 weeks, LOXL3 was abundant throughout the PDL and showed strong expression in both the diabetic and control groups (Figure 4F–I); however, the expression was significantly higher in the diabetic group (Figure 4J; diabetic group: MD=0.0749±0.003 and MD=0.0646±0.0063; p<0.05).

After 9 and 16 weeks, LOX expression in the diabetic and control groups was evaluated respectively (Figure S1A–D; Figure S1F–I). LOX expression at 9 weeks (control group: MD=0.0449±0.0080; diabetic group: MD=0.0469±0.0039) and 16 weeks (control group: MD=0.0456±0.0047; diabetic group: MD=0.0449±0.0041) was observed and the differences of LOX positive expression were insignificant (Figures S1E and S1J; p>0.05). n=5, repeated three times

After 9 and 16 weeks, LOXL1 expression in the diabetic and control groups was evaluated respectively (Figure S2A–D; Figure S2F–I). LOXL1 expression at 9 weeks (control group: MD=0.0483±0.0026; diabetes group: MD=0.0456±0.0019) and 16 weeks (control group: MD=0.0567±0.0092; diabetes group: MD=0.0532±0.0044) was observed and the differences of LOXL1 positive expression were insignificant (Figures S2E and S2J; p>0.05). n=5, repeated three times

After 9 and 16 weeks, LOXL2 expression in the diabetic and control groups was evaluated respectively (Figure S3A–D; Figure S3F–I). LOXL2 expression at 9 weeks (control group: MD=0.0461±0.0056; diabetes group: MD=0.0448±0.0078) and 16 weeks (control group: MD=0.0487±0.0049; diabetes group: MD=0.0451±0.0078) was observed and the differences of LOXL2 positive expression were insignificant (Figures S3E and S3J; p>0.05). n=5, repeated three times

## Discussion

T2DM is a chronic metabolic disorder characterized by chronic hyperglycemia and insulin resistance.^18^ Numerous diseases are associated with T2DM,^19^ such as cardiovascular atherosclerosis,^20^ osteoarthritis,^21^ chronic kidney disease,^22^ and metabolic syndrome.^23^ In clinical dentistry, it has been observed that, when compared with non-T2DM patients, T2DM patients have a higher risk of gingival inflammation and periodontal disease.^24^ Borgnakke, et al.^25^ (2013) and Kim, et al.^26^ (2014) showed a strong correlation between periodontitis and T2DM. However, the precise mechanism remains unclear.

Periodontitis is a common oral disease associated with chronic pain. Without systemic treatment, the periodontal condition gradually becomes severe, which can lead to excessive alveolar bone resorption,^27,28^ receding gums, and tooth loss.^29^ Otomo-Corgel, et al.^30^ (2012) and Borgnakke, et al.^25^ (2013) showed that T2DM patients have distinct microvascular lesions in gingival vessels and a significantly thickened basement membrane. In this study, after 16 weeks, abnormalities in fibroblasts and collagen fibers in diabetic rats were observed. Moreover, they also presented aggressive alveolar bone destruction,^26^ which may be due to the gradual severity of periodontal lesions over time. It highlights the importance of early diagnosis of periodontal diseases induced by diabetes.

Studies showed a positive correlation between blood glucose levels and the occurrence and progression of periodontitis.^31^ Mealey^32^ (1996) showed that the prevalence and severity of periodontitis is higher in T2DM patients when compared with T1DM patients. The PDL is susceptible to bacterial infections due to chronic hyperglycemia or poor long-term blood glucose control.^33-35^ The degree of periodontal tissue damage in T2DM patients is significantly reduced when blood glucose was well controlled.^32^ Our previous study showed that the blood glucose of rats in the diabetic group was significantly higher when compared with the control group after both 9 and 16 weeks.^7^ Interestingly, significant histopathological changes were observed at 16 weeks. This confirmed that diabetes-induced periodontal pathological changes are indistinguishable. Therefore, early and positive blood glucose control is of utmost importance for the intervention of periodontal diseases in T2DM patients.

Changes in collagen metabolism can lead to periodontal diseases and other abnormalities in T2DM patients, such as oral ulceration and damaged wound healing.^36^ Arita, et al.^37^ (2016) showed that alveolar bone remodeling can also be affected in diabetic rats, resulting in impaired orthodontic tooth movement. In this study, obvious abnormal deformation and disordered arrangement of the PDL were observed in diabetic rats after 16 weeks. Microscopic observation showed changes in the morphology and structure of the PDL, such as bending, overlapping, and high disorder, which suggests excessive cross-linking of collagen fibers.^38^ LOXs are widely distributed in organs and organ function may be largely regulated by their activity.^10^ LOXL3 may be related to human Stickler syndrome^39^ and high myopia.^40^Moreover, LOXL3 knockout mice were found to have a cleft palate and decreased collagen density in the palatal region of mouse embryos,^40^ suggesting that abnormal LOXL3 expression may be associated with oral diseases. Therefore, we focused on LOXL3 as our target enzyme and our findings were in accordance with the changes in the PDL, as LOXL3 increased in diabetic rats. LOXL3 plays an indispensable role in degenerative changes associated with T2DM, including abnormal deformation and disordered arrangement of the periodontal tissue. This shows that LOXL3 could be used as an indicator for the early diagnosis of periodontal diseases induced by diabetes. It is also very significant for the prevention and treatment of periodontal diseases in T2DM patients.

The significant upregulation of LOXL3 expression in the PDL of diabetic rats shows that LOXL3 may be a key factor in the occurrence and development of periodontal diseases in T2DM patients. In this study, we initially observed changes in LOX expression in the periodontal tissue *in vivo*, but lacked molecular diagnosis *in vitro*. Further studies are needed to confirm the function of LOXs, especially LOXL3, in the occurrence and development of periodontal diseases induced by diabetes.

## Conclusion

In conclusion, the abnormally increased LOXL3 expression in diabetic rats affected the PDL. Our study showed that LOXL3 could be a crucial factor in the early diagnosis of histopathological changes in periodontal diseases of T2DM patients. Further *in vivo* studies are required to clarify these mechanisms.
